# Impact of *IL28B*-Related Single Nucleotide Polymorphisms on Liver Transient Elastography in Chronic Hepatitis C Infection

**DOI:** 10.1371/journal.pone.0080172

**Published:** 2013-11-14

**Authors:** Magdalena Ydreborg, Johan Westin, Karolina Rembeck, Magnus Lindh, Hans Norrgren, Anna Holmberg, Rune Wejstål, Gunnar Norkrans, Kristina Cardell, Ola Weiland, Martin Lagging

**Affiliations:** 1 Department of Infectious Diseases/Virology, Institute of Biomedicine, University of Gothenburg, Gothenburg, Sweden; 2 Department of Infectious Diseases, Skåne University Hospital, Lund, Sweden; 3 Department of Infectious Diseases, Linköping University Hospital, Linköping, Sweden; 4 Department of Infectious Diseases, Karolinska Institutet, Karolinska University Hospital, Karolinska, Sweden; St. Louis University, United States of America

## Abstract

**Background and Aims:**

Recently, several genome-wide association studies have revealed that single nucleotide polymorphisms (SNPs) in proximity to *IL28B* predict spontaneous clearance of hepatitis C virus (HCV) infection as well as outcome following pegylated interferon and ribavirin therapy among genotype 1 infected patients. Additionally the presence of the otherwise favorable *IL28B* genetic variants in the context of HCV genotype 3 infection reportedly entail more pronounced liver fibrosis and steatosis. The present study aimed to evaluate the impact of *IL28B* SNP variability on liver stiffness as accessed by transient elastography.

**Methods:**

Seven hundred and seventy-one Swedish HCV infected patients sequentially undergoing liver stiffness measurement by means of Fibroscan® in the context of a real-life trial had samples available for *IL28B* genotyping (*rs12979860*) and HCV genotyping.

**Results:**

CC*_rs12979860_* was more common among HCV genotype 2 or 3 infected treatment-naïve patients than among those infected with genotype 1 (P<0.0001). Additionally CC*_rs12979860_* among HCV genotype 3 infected patients was associated with higher liver stiffness values (P = 0.004), and higher AST to platelet ratio index (APRI; p = 0.02) as compared to carriers of the T allele. Among HCV genotype 1 infected patients, CC*_rs12979860_* was significantly associated with higher viral load (P = 0.001), with a similar non-significant trend noted among HCV genotype 3 infected patients.

**Conclusion:**

This study confirms previous reports that the CC*_rs12979860_* SNP is associated with more pronounced liver pathology in patients chronically infected with HCV genotype 3 as compared to genotype 1, suggesting that *IL28B* genetic variants differently regulates the course of HCV infection across HCV genotypes.

## Introduction

Hepatitis C virus (HCV) infects 170 million people worldwide [1] and is a leading cause of chronic hepatitis, cirrhosis, and hepatocellular carcinoma [2]. Recently, several genome-wide association studies have revealed that single nucleotide polymorphisms (SNPs) in the *19q13* region, in close proximity to three genes (*IL28A*, *IL28B*, and *IL29*) encoding cytokines of the interferon-λ (*i.e.* type III interferon) family, predict spontaneous clearance of HCV infection [3], [4] as well as sustained virological response (SVR) following pegylated-interferon (peg-IFN) and ribavirin therapy among patients infected with HCV genotype 1 [3], [5], [6], [7].

The C allele at *rs12979860* is associated with higher viral load [5], [8], [9], which otherwise is an established negative predictor of response to IFN-α and ribavirin therapy [10], [11], [12]. Additionally, these polymorphisms are strongly associated with the first phase viral decline (*i.e.* reduction of HCV RNA during the first days of treatment which is assumed to result from the blocking of the production or release of hepatitis C virions [13], [14]), irrespective of HCV genotype [9], [15], [16]. Among HCV genotype 1 infected patients this translates into higher frequencies of achieving both rapid virological response (RVR) and SVR among carriers of the favorable SNP alleles [9], [15], and concomitant assessment of pretreatment levels of systemic IP-10 augments the predictive value of *IL28B* genetic variants [17], [18].

Somewhat counterintuitive are the multiple reports that C allele carriage at *rs12979860* is more common in Caucasians infected with HCV genotype 2 or 3 than with genotype 1 [9], [18]. In the setting of therapeutic intervention for HCV genotype 2 or 3, uncertainty prevails regarding the benefit of favorable *IL28B* allele carriage. Sarrazin *et al.* reported increased SVR rates following therapy among HCV genotype 2 or 3 infected Caucasian CC *rs12979860* carriers as compared to carriers of the T allele [19]. In contrast Mangia *et al.* noted an association between *IL28B* genotype and SVR only among HCV genotype 2 or 3 infected patients failing to achieve RVR [20]. Yu *et al.* reported a significantly higher rate of achieving RVR but not SVR among Asian homozygous TT *rs8099917* carriers (*i.e.* the favorable genotype for this SNP) infected with HCV genotype 2 [21] and Moghaddam *et al.* noted similar results among HCV genotype 3 infected Caucasian CC *rs12979860* carriers [22] as did Scherzer *et al.* [23]. In contrast, Lindh *et al.* [16] and Stenkvist *et al.* [24] reported no impact of CC *rs12979860* carriage on the likelihood of achieving either RVR or SVR, in spite of a steeper first phase decline in HCV RNA among Scandinavian genotype 2/3 infected patients, possibly secondary to a higher baseline viral load.

In a study of Japanese patients infected with HCV genotype 1 or 2, those homozygous for the *IL28B* favorable allele had significantly more severe inflammatory activity, and a higher proportion of these patients had fibrosis stage F2-4 as compared with F0-1 [25]. In contrast, among HCV genotype 1 infected American patients, CC carriers at *rs12979860* reportedly was associated with a lower prevalence of steatosis [26]. Among HCV genotype 3 infected patients of Scandinavian descent, CC carriers at *rs12979860* had significantly higher normalized alanine aminotransferase (ALT) levels as well as aspartate aminotransferase platelet ratio index (APRI) than T allele carriers indicating more pronounced inflammation and fibrosis [22]. Similarly when evaluating liver biopsies from HCV genotype 3 infected patients, *IL28B* genetic variants that otherwise are linked with more favorable therapeutic outcome were associated with more pronounced inflammation, steatosis, and fibrosis [27], [28], [29]. Among 1483 predominantly HCV genotype 1 infected patients, 276 of whom had paired liver biopsies, CC carriers at *rs12979860* had greater hepatic necroinflammation, higher ALT, and worse clinical outcome, but not greater fibrosis progression, although this latter finding may have been secondary to the relatively short time that elapsed between biopsies (median 4 years) [30]. Following HCV recurrence among 54 liver transplant recipients, a non-significant trend towards milder fibrosis was noted among CC *rs12979860* carriers possibly secondary to better therapeutic response [31]. Subgrouping by HCV genotype, however, was not reported in this latter study.

The aim of the present study was thus to evaluate the impact of *IL28B* SNP variability on liver damage evaluated by liver stiffness measurement in the context of a real-life trial for sequential patients with HCV infection undergoing routine evaluation.

## Materials and Methods

### Study participants

**Figure 1:**
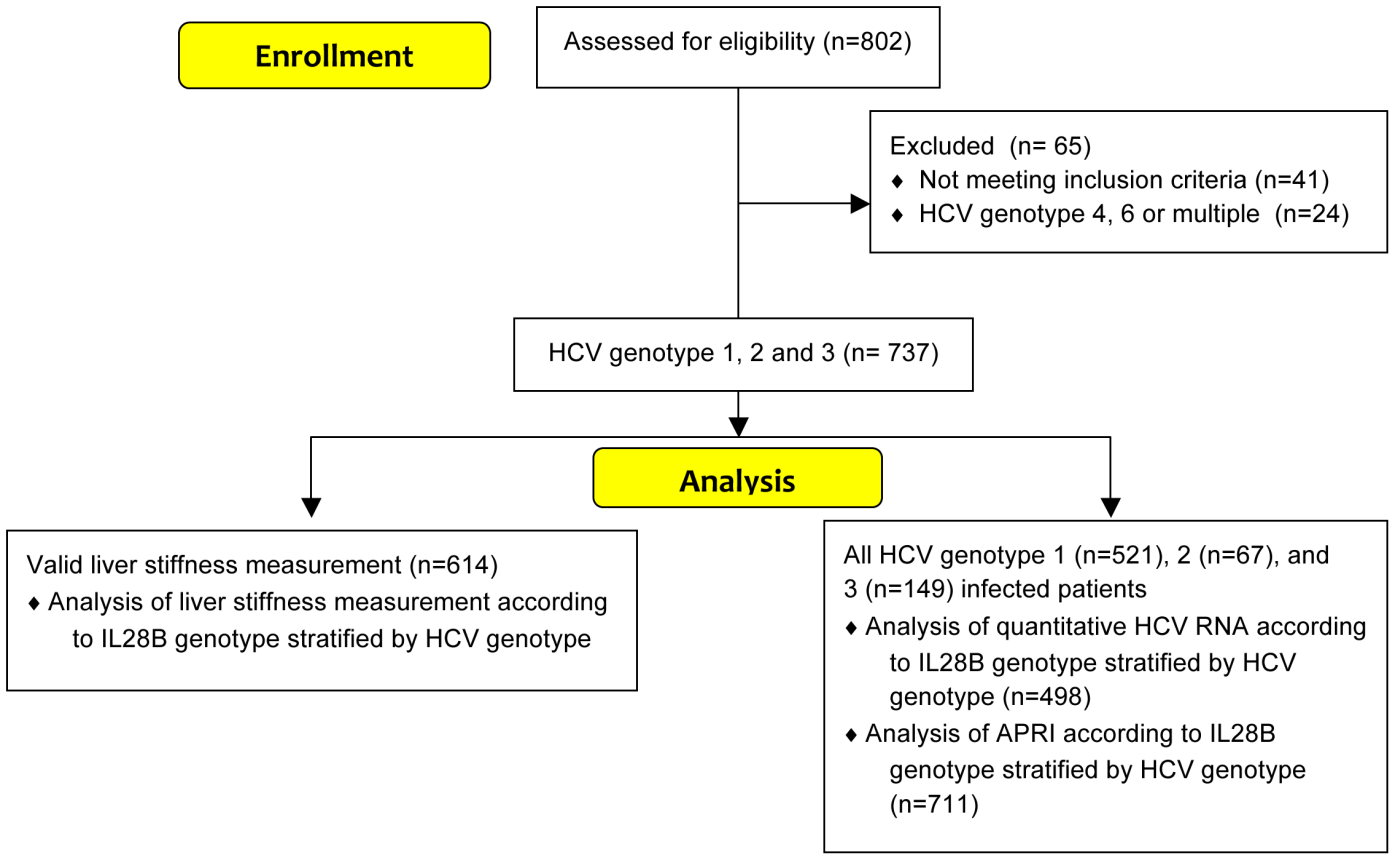
Study enrollment and disposition of patients.

Eight hundred and two sequential Scandinavian HCV infected patients undergoing routine clinical liver stiffness measurement from 2008 to 2012 were recruited at four University Hospitals in Sweden, and genotyped for *IL28B* (*rs12979860*) (enrollment and disposition of patients detailed in Figure 1). Forty-one of the evaluated patients did not fulfill the inclusion criteria (HCV-RNA was not detectable at time of liver stiffness measurement, or unavailable samples for *IL28B* or HCV genotyping) and thus were excluded. One patient with genotype 6, 21 patients with genotype 4, and two patients co-infected with more than one HCV genotype were also excluded leaving a study cohort of 737 patients, of whom 614 had valid liver stiffness measurements (characteristics detailed in Table 1). No patients had detectable hepatitis B surface antigen (HBsAg) or anti-HIV antibodies. Demographic and clinical data were gathered from medical charts and anonymously registered in a joint database. Information regarding possible previous episodes of antiviral treatment was available for 708 patients (of whom 590 had an interpretable liver stiffness measurement) where 21% (n = 150) of the patients were treatment experienced, without having achieved SVR. No patient was on treatment at the time of evaluation. Data regarding alcohol consumption or race was not available, although the overwhelming majority of patients are likely to be Caucasians of Scandinavian origin.

**Table 1:** Baseline characteristics of patients with a valid liver stiffness measurement.

	Genotype 1	Genotype 2	Genotype 3	P-value
	n = 445	n = 44	n = 125	
Gender (male/female)^a^	285/160	28/16	73/52	n.s
Age (years)^b^	52 (44-58)	56 (49-64)	47 (41-54)	<0.0001
Duration of infection (years)^b.c^	30 (22-35)	34 (25-38)	24 (11-30)	<0.0001
BMI (kg/m^2^)^b,^ d	26 (23-28)	29 (26-30)	24 (23-26)	0.02
Mode of transmission^e^				n.s
Intravenous drug use	49%	52%	64%	
Blood transfusion	21%	21%	15%	
Other/unknown	30%	27%	21%	

Values are expressed as n^a^, median (IQR)^b^ or percentage^e^

c n = 318, n = 34, and n = 91 for genotype 1, 2, and 3 respectively

d n = 152, n = 6, and n = 51 for genotype 1, 2, and 3 respectively

### *IL28B* genotyping

SNP *rs12979860* was determined in plasma by allelic discrimination using Taqman MGB (minor groove binding) probes. The following primers and probes were used: *rs12979860*: Forward, GTGCCTGTCGTGTACTGAACCA, Reverse, AGCGCGGAGTGCAATTCA, Probe_C, FAM-CCTGGTTCGCGCCTT-MGB, Probe_T, VICCCTGGTTCACGCCT-MGB. All SNPs were in Hardy-Weinberg equilibrium. SNP *rs12979860* has previously been reported to have a stronger association with both first phase decline and SVR than *rs8099917* and *rs12980275* among Caucasian HCV infected patients, and was thus analyzed in the present study [9].

### HCV RNA quantification

Plasma was obtained using PPT-tubes and HCV RNA was determined by RT-PCR of plasma using Cobas AmpliPrep/COBAS TaqMan HCV Test (Roche Diagnostics, Branchburg, NJ), which quantifies HCV RNA with a limit of detection of ≤15 IU/mL.

### Liver stiffness measurement

Liver stiffness measurement was performed using the Fibroscan® device (EchoSens, Paris, France). Details of the technical background and the examination procedure have been described previously [32]. The results were expressed as median values in kilo Pascal (kPa). Procedures with ten valid measurements and a success rate (the number of valid measurements divided by the number of all measurements) of at least 60% were considered reliable. In addition, the median value of successful measurements was considered representative for the liver stiffness in a given patient if the interquartile range (IQR) of the examinations was less than 30% of the median [33], [34].

### Fibrosis index

APRI was calculated as the ratio of normalized aspartate aminotransferase, *i.e.* value divided by the upper limit of normal, to the platelet count as previously detailed [35].

### Statistical methods

Wilcoxon-Mann-Whitney U-test, Kruskal-Wallis test, and Chi squared (χ^2^) tests were utilized to evaluate relationships between groups. Multivariate analysis of potential fibrosis predictors was made by General Linear Model. Logarithmic transformation was used for quantitative data with skewed distribution and subjects were stratified according to genotype. The following variables were included in the analysis: age, gender, normalized ALT and IL28B genotype with median value of Liver Stiffness Measurement as dependent variable. A model including BMI was rejected since this resulted in too few observations in non-genotype 1 patients to perform multivariate analysis. All statistical analyses were performed using IBM SPSS Statistics version 19.0 software package (IBM Corporation, Somers, NY). All reported p-values are two-sided, and p-values <0.05 were considered significant. IL28B SNPs comparisons were made by stratifying patients according to rs12979860CC and *rs12979860*CT/TT genotypes.

### Ethical Statement

The treatment study conformed to the guidelines of the 1975 Declaration of Helsinki and was approved by the Regional Ethical Review Board in Gothenburg (Regionala etikprövningsnämnden i Göteborg). Written informed consent was obtained from each participating patient.

## Results

**Figure 2:**
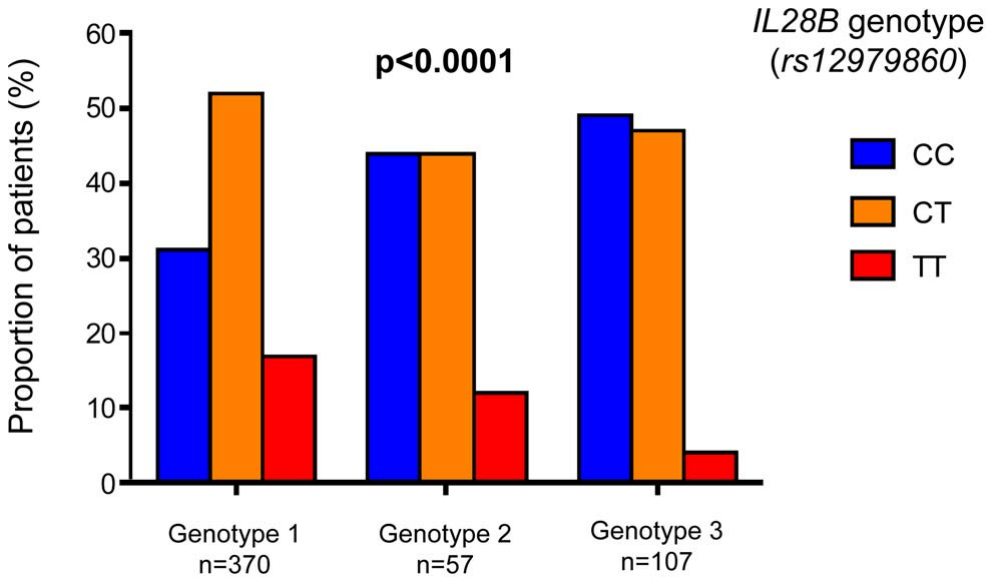
Frequency distribution of *IL28B* variants in relation to HCV genotypes 1-3 among treatment-naïve patients. Chi-squared (*χ* ^2^)-test was used to compare differences in distribution.

The majority of patients were male (62%) and infected with HCV genotype 1 (69%). When comparing HCV genotype 1 and 3 infected patients, they differed significantly. The HCV genotype 1 patients were older (median age 53 vs. 47 years for genotype 1 and 3 respectively, p<0.0001), had a longer duration of infection (median 30 vs. 25 years for genotype 1 and 3 respectively, p<0,0001), had higher BMI (26 vs. 25 kg/m^2^ for genotype 1 and 3 respectively, p = 0.04), and were less likely to have been infected through intravenous drug use (50% vs. 64% genotype 1 and 3 respectively, p = 0.03). A strong association was noted between the distribution of HCV genotypes and *IL28B* SNP variants (P<0.0001; Figure 2), with CC at *rs12979860* being significantly more common in treatment-naïve patients with HCV genotype 2 or 3 infection than genotype 1. Treatment-experienced patients were excluded from this latter analysis in order to avoid potential bias resulting from differing treatment response.

A valid liver stiffness measurement was obtained in 614 patients (83%). In general patients with invalid examinations were older than those with valid (median age 54 and 52 years, p = 0.004), and had significantly higher body-mass index (BMI) (28 and 25 kg/m^2^, p<0.0001). These findings were in line with a previous reported 5-year prospective study of 13,369 liver stiffness measurements, where unreliable results were noted in nearly one of five examinations, with obesity and old age being main causes [36].

**Figure 3:**
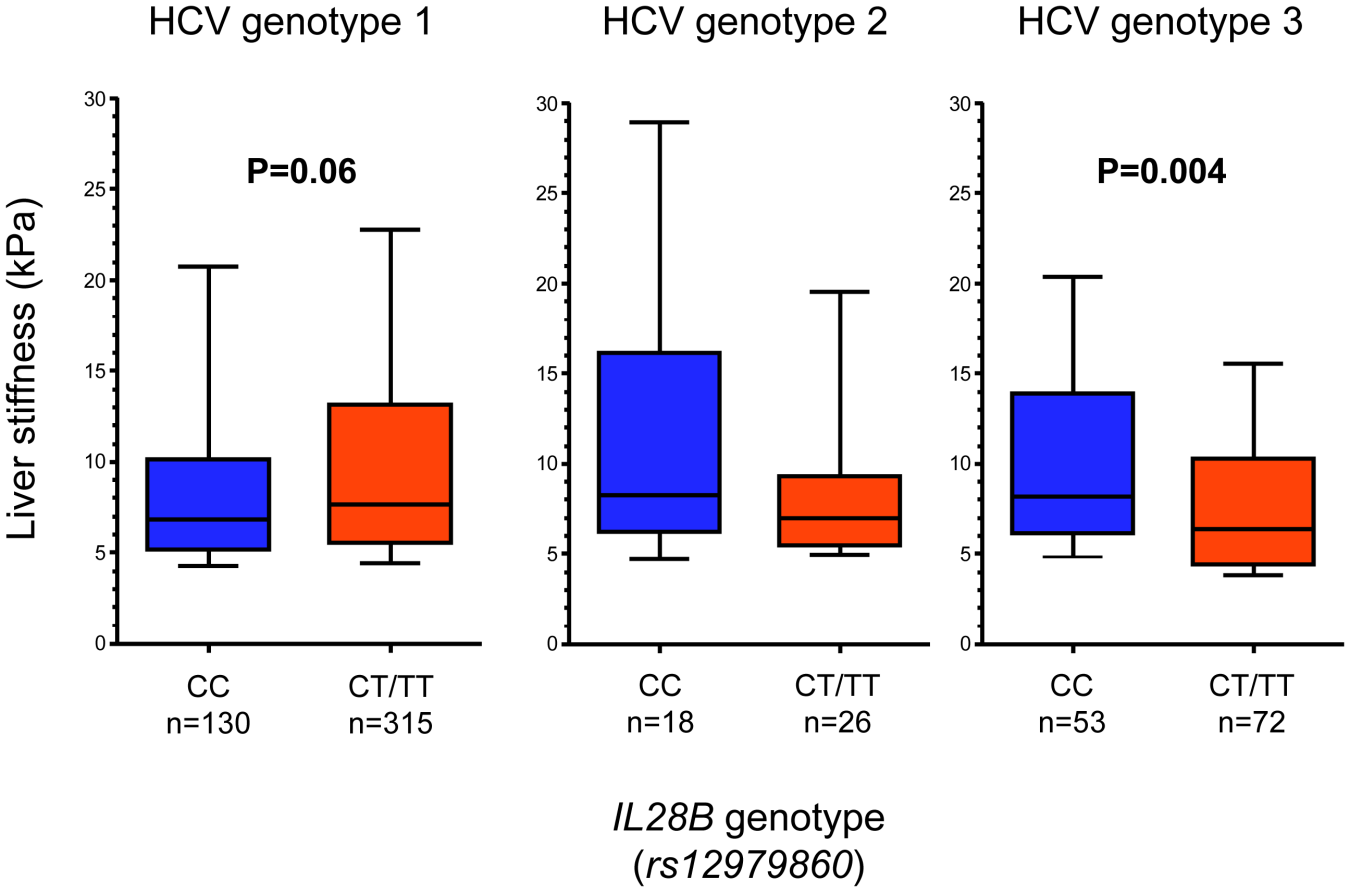
Tenth, 25^th^, 50^th^, 75^th^, and 90^th^ percentiles of liver stiffness measurement level in relation to *IL28B* variants for genotypes 1, 2 and 3.

**Figure 4:**
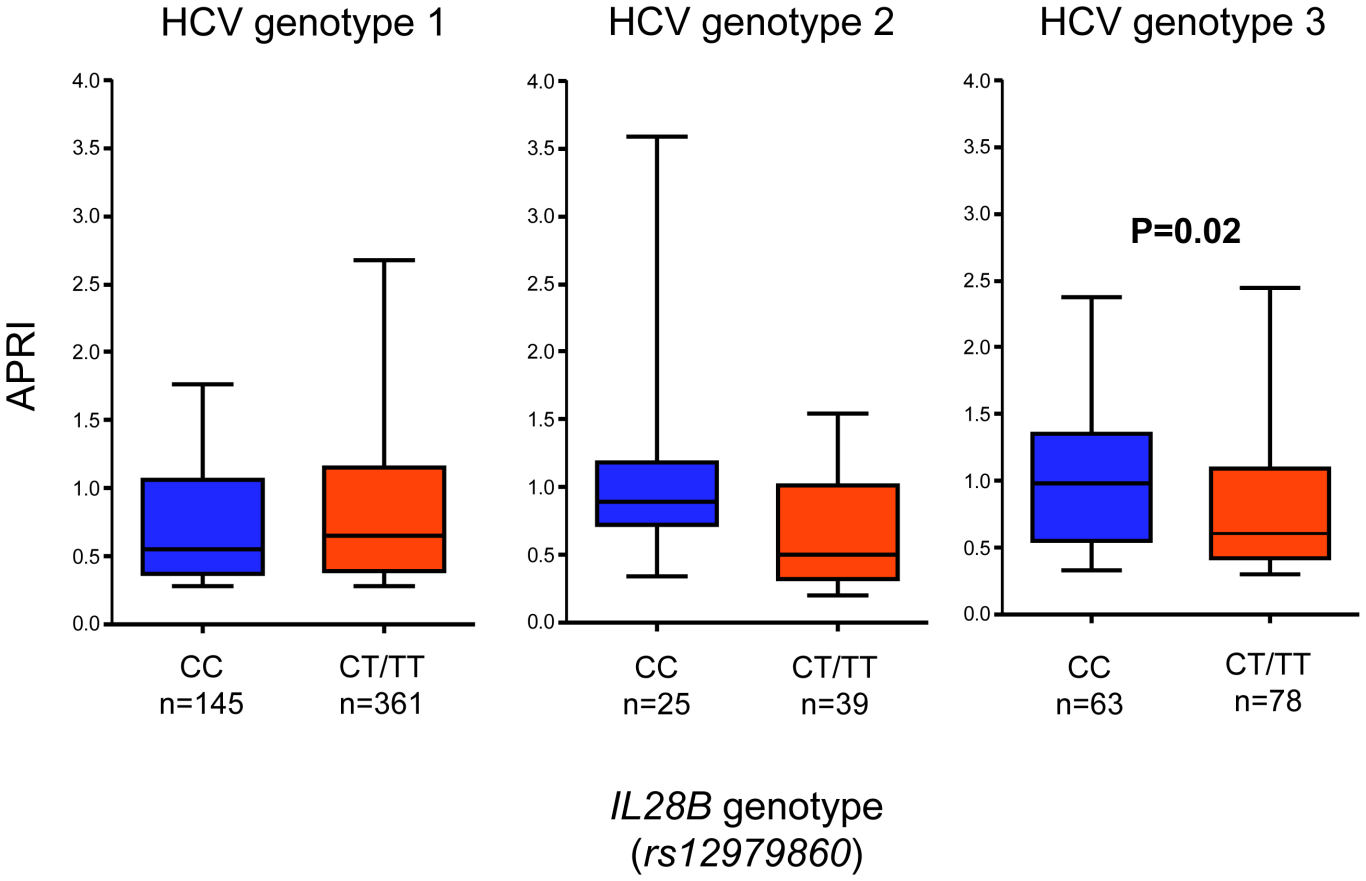
Tenth, 25^th^, 50^th^, 75^th^, and 90^th^ percentiles of APRI score in relation to *IL28B* variants for genotypes 1, 2 and 3.

Among HCV genotype 3 infected patients with CC at *rs12979860*, significantly higher liver stiffness values (median 8.2 vs. 6.4 kPa for CC and CT/TT respectively, P = 0.004; Figure 3) as well as APRI (median 1.0 vs. 0.6 for CC and CT/TT respectively, P = 0.02; Figure 4), were noted as compared to T*_rs12979860_* allele carriers. Conversely, among HCV genotype 1 infected patients with CC genotype, a non-significant trend towards lower liver stiffness values and APRI were noted. There were no significant differences in age, gender, BMI or duration of infection between CC and CT/TT carriers in neither genotype 1 nor genotype 3 infected patients (Table 2). These results were unchanged when patients with unreliable liver stiffness measurements were excluded. No significant associations were observed among the 67 HCV genotype 2 infected patients, although a trend was noted towards slightly more pronounced liver pathology among CC carriers (median liver stiffness 9.2 vs. 7.0 kPa for CC and CT/TT respectively, P = 0.13), (median APRI 0.8 vs. 0.5 for CC and CT/TT respectively, P = 0.19). All of the abovementioned results remained unchanged if treatment-experienced patients were excluded from the analyses. No association was noted between alanine aminotransferase (ALT) and *IL28B* genetic variants.

**Table 2:** Baseline characteristics according to HCV genotype and IL28 B genetic variations (*rs12979860*).

	Genotype 1			Genotype 3		
	CC	CT/TT		CC	CT/TT	
	n = 151	n = 370		n = 67	n = 82	
Age (years)^a^	52 (43-58)	53 (46-58)	n.s	48 (41-54)	47 (42-55)	n.s
Gender (male/female)^b^	96/55	240/130	n.s	38/29	45/37	n.s
BMI (kg/m^2^)^a,^ c	26 (23-28)	26 (24-29)	n.s	26 (23-27)	24 (23-26)	n.s
Duration of infection (years)^a,^ d	30 (21-36)	31 (22-36)	n.s	26 (15-31)	24 (11-31)	n.s

Values are expressed as median (IQR)^a^ or n^b^

c n = 173 genotype 1, n = 58 genotype 3

d n = 373 genotype 1, n = 109 genotype 3

**Figure 5:**
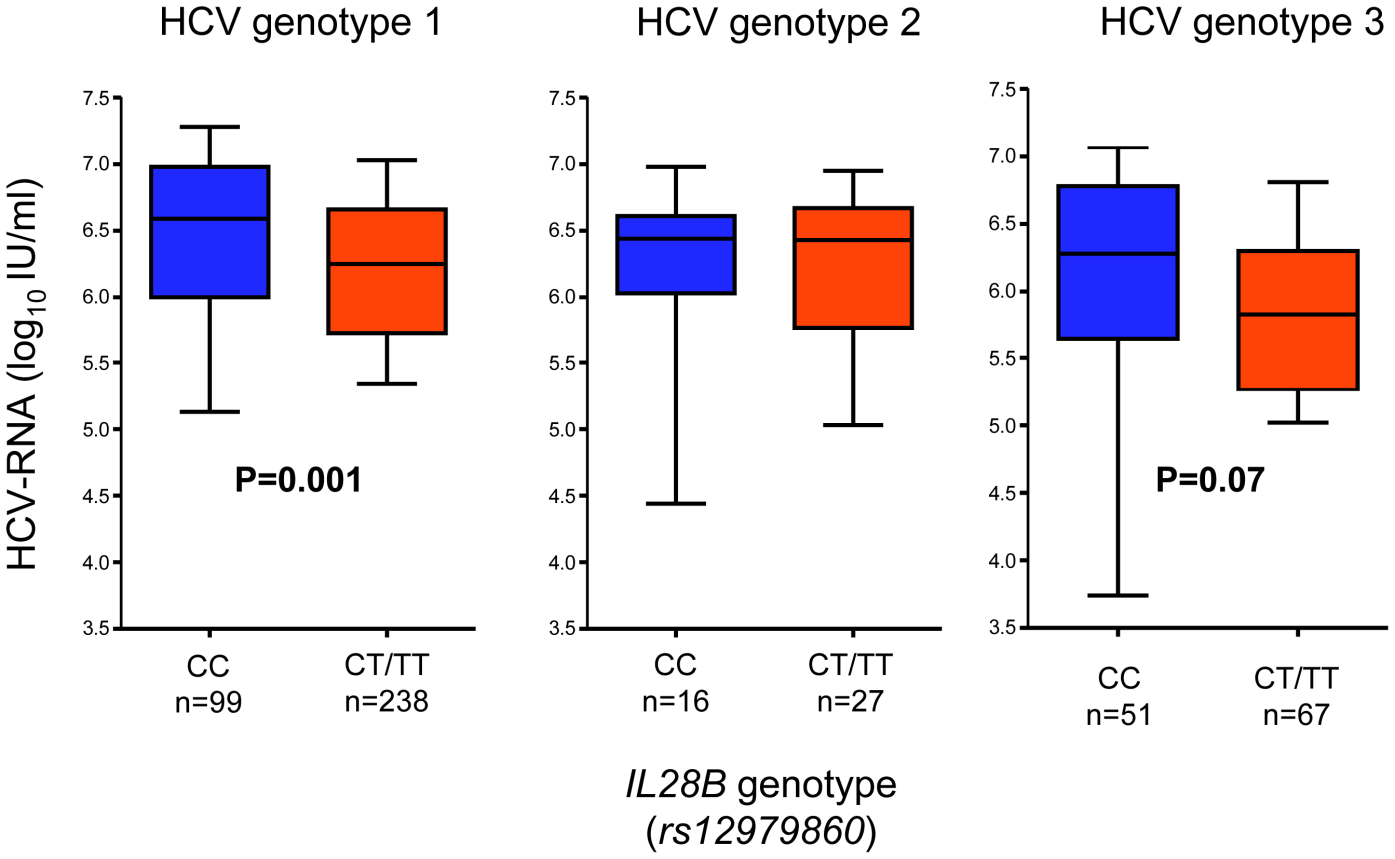
Tenth, 25^th^, 50^th^, 75^th^, and 90^th^ percentiles of HCV RNA level in relation to *IL28B*.

The HCV genotype 1 infected homozygous CC carriers had significantly higher viral load (median 6.6 and 6.2 log_10_ IU/mL for CC and CT/TT respectively, P = 0.001; Figure 5) with similar non-significant trend noted among HCV genotype 2 and 3 infected patients.

The following variables remained independently predictive in multivariate analysis of greater liver stiffness in HCV genotype 1 infected patients: older age (P<0.001), higher ALT (P<0.0001), and male gender (P = 0.011). For HCV genotype 3 infected patients, older age (P<0.0001), higher ALT (P = 0.001), CC at *rs12979860* (P = 0.017), and male gender (P = 0.029) were independently predictive of more pronounced liver stiffness. HCV genotype 2 infected patients could not be evaluated in multivariate analysis due to the small sample size.

## Discussion

This study confirms previous reports that CC*_rs12979860_* carriage is associated with more pronounced liver pathology in patients chronically infected with HCV genotype 3 as compared to genotype 1. Abe *et al*. reported that among Japanese patients infected with HCV genotype 1 or 2, patients with homozygous carriage of the *IL28B* major allele had significantly more severe inflammatory activity and fibrosis, indicating that this SNP genotype may not be beneficial outside the context of therapeutic intervention [25]. Similarly Bochud *et al.* [37] analyzed the association between *IL28B* polymorphisms and liver histology among 1527 chronically HCV-infected Caucasian patients and noted that the rare *IL28B* G*_rs8099917_* allele, which has been associated with poor response to therapy, entailed less hepatic inflammation and fibrosis. When stratifying for HCV genotype, important differences were noted, and the findings were statistically significant for genotype 3 only. In agreement with the findings of Bochud *et al.*, we recently presented results from a pegylated interferon-α2a and ribavirin trial for treatment naïve HCV genotype 2/3 patients (NORDynamIC study) [38]. In this trial, which included 314 Caucasian patients who were evaluated for *IL28B* polymorphisms, pretreatment liver biopsies were mandatory and centrally evaluated for liver fibrosis and inflammation using the Ishak protocol as well as steatosis. The *IL28B* G*_rs8099917_* allele was significantly associated with milder fibrosis among HCV genotyped 3 infected patients, with a similar non-significant trend observed for *IL28B* T*_rs12979860_*, but not for genotype 2 [27], [28]. Similarly, *IL28B* T*_rs12979860_* carriage in HCV genotype 3 infected patients was associated with less steatosis, whereas *IL28B* G*_rs8099917_* carriage in HCV genotype 2 was associated with less steatosis.

Liver pathology in the present study was evaluated by means of liver stiffness measurement rather than liver biopsy. Thus it was not possible to ascertain which histopathological components contributed to the elevated measurements among HCV genotype 3 infected CC carriers, although the concomitantly elevated APRI suggests that more pronounced fibrosis weighed in. It should be noted that elevated ALT reportedly confounds the use of transient elastography among HCV infected patients [39]. However, in the present study ALT was not associated with *IL28B* genetic variants, and in the multivariate analysis among HCV genotype 3 infected patients, both higher ALT and *IL28B* CC*_rs12979860_* carriage were independently predictive of the elevated liver stiffness measurement. Although transient elastography has a high degree of accuracy and reproducibility in prediction of liver fibrosis in the context of HCV infection, there are conflicting reports as to the impact of steatosis on Fibroscan® measurements. Arena *et al.* observed no influence of steatosis on transient elastography [40], whereas Sanchez-Conde *et al.* [41] and Boursier *et al.* [42] noted significant associations. However, in the latter two studies the influence of steatosis was noted predominately among patients with high-grade steatosis.

It is unclear how CC genotype carriage, in the context of HCV genotype 3 infection, may induce more pronounced liver pathology. Rembeck *et al.* previously suggested that the underlying mechanism of action might be secondary to higher baseline viral loads [27], [28], [29], although in the present study no significant association was noted between viral load and liver stiffness. Elevated HCV RNA levels in HCV genotype 3 infection have previously been reported to be associated with the increased presence and severity of steatosis [43], [44], which in turn entails accelerated fibrosis progression [45], suggestive of a cytopathic effect of HCV genotype 3 virus. On a similar note, it was recently reported that the cumulative mortality of HCV genotype 3 infected US Department of Veterans Affairs (VA) patients failing to achieve SVR after therapy was higher than among non-SVR patients infected with genotypes 1 or 2 [46]. How the HCV genotype 3 virus exerts this cytopathic effect remains to be determined, though previously it has been hypothesized that the greater propensity for development of steatosis, than observed for other HCV genotypes [43], [45], may be secondary to a greater impairment of lipid export from infected hepatocytes [47], [48] possibly mediated by inhibition of microsomal triglyceride transfer protein (MTP) [49], [50] or due to increased availability of free fatty acids by reduced oxidation or by increased *de novo* synthesis [51], [52], [53], [54] mediated by the HCV genotype 3 core protein.

Due to the retrospective design, details regarding alcohol intake were not recorded. Similarly, according to clinical routine, information regarding ethnicity was also not recorded in medical charts at the participating centers. However, when considering the local populations of HCV-infected individuals in Sweden, it is reasonable to assume that the overwhelming majority of patients included were Caucasians of Scandinavian origin.

Our finding that homozygous CC at *rs12979860* was significantly more common in the setting of treatment-naïve HCV genotype 2 or 3 infection than genotype 1 corroborates previous reports [8], [18]. Indeed, the proportion of CC at *rs12979860* among HCV genotype 2 and 3 infected patients (40% and 45%, respectively) in our study is similar to the reported prevalence in HCV uninfected Caucasians (∼40%), suggesting that this SNP genotype may be less beneficial following exposure to HCV genotype 2 or 3 as compared to genotype 1.

In conclusion, the present study demonstrated an association between CC carriage at *rs12979860* and more pronounced liver stiffness values and APRI among HCV genotype 3 infected patients. In this light, analysis of *IL28B* genotype may be beneficial among HCV genotype 3 infected patients so as to encourage homozygous CC *rs12979860* carriers to initiate therapy. Additionally, the finding that *IL28B* variability did not significantly impact on liver stiffness measurement among HCV genotype 1 and 2 infected patients and that CC *rs12979860* was more common among genotype 2/3 infected patients, implies that *IL28B* may differentially regulate the course of HCV infection across genotypes.
